# Effect of Shenmai injection on cognitive function after cardiopulmonary bypass in cardiac surgical patients: a randomized controlled trial

**DOI:** 10.1186/s12871-018-0604-7

**Published:** 2018-10-11

**Authors:** Lei Chen, Liangrong Wang, Qian Zhuo, Qiong Zhang, Feifei Chen, Liling Li, Lina Lin

**Affiliations:** 1 Department of Anesthesiology, The First Affiliated Hospital of Wenzhou Medical University, Wenzhou, Zhejiang Province China; 2 Wenzhou People’s Hospital, Wenzhou, Zhejiang Province China

**Keywords:** Cardiopulmonary bypass, Cognitive dysfunction, Shenmai injection, Cerebral protection

## Abstract

**Background:**

Postoperative cognitive dysfunction (POCD) is a common complication after cardiac surgery that influences the clinical outcomes and quality of life of patients. This study aimed to evaluate the effects of Shenmai injection (SMI) on POCD of patients who underwent cardiac valve replacement under cardiopulmonary bypass (CPB).

**Methods:**

This prospective, randomized, controlled trial was conducted from September 2014 to January 2017. Eighty-eight patients receiving cardiac valve replacement under CPB were randomized into the control (C) or the SMI (S) group. SMI (0.6 mL/kg) was administered intravenously from the time of anesthesia induction to the beginning of CPB. Cognitive function was assessed at 3 days before surgery and 3 days, 7 days, and 1 month after surgery using the Beijing version of the Montreal Cognitive Assessment (MoCA-BJ) score. The serum levels of neuroglobin (Ngb), hypoxia-inducible factor-1α (HIF-1α), and neuron-specific enolase (NSE) were measured at 30 min after induction (T_0_), immediately after the endonasal temperature rewarmed to 36 °C (T_1_), and 1 h (T_2_), 6 h (T_3_), 24 h (T_4_), 48 h (T_5_), and 72 h (T_6_) after CPB.

**Results:**

Compared with the baseline values at T_0_, the serum Ngb levels in group C were significantly decreased at T_1–2_ and then increased at T_3–6_, while the levels in group S were decreased at T_1–2_ and increased at T_4–6_, compared to group C (*p* < 0.05). The serum HIF-1α levels at T_1–4_ and the serum NSE levels at T_1–6_ were significantly increased in both groups (*p* < 0.05). The serum levels of Ngb at T_3_, HIF-1α at T_1–3_, and NSE at T_3–4,6_ were lower in group S, compared to group C (*p* < 0.01). The MoCA-BJ scores were decreased at 3 and 7 days after surgery in both groups, and the MoCA-BJ scores in group S were higher than those in group C at 3 and 7 days after surgery (*p* < 0.01).

**Conclusion:**

Cognitive function is impaired postoperatively in patients who have undergone cardiac valve replacement under CPB. In addition, treatment with the traditional Chinese medicine SMI decreases the serum levels of Ngb, HIF-1α, and NSE as well as attenuates cognitive dysfunction.

**Trial registration:**

This trial was registered with Clinicaltrials.gov as ChiCTR-TRC-14004373 on March 11, 2014.

## Background

Postoperative cognitive dysfunction (POCD) is considered as a common complication of cardiovascular surgery and causes several adverse effects, such as a delayed long-term recovery, a reduced quality of life, and an increased mortality rate [1, 2]. Cerebral embolization, systemic inflammatory response, and low cerebral oxygenation to cerebral hypoperfusion are usually implicated as potential factors for short-term POCD after cardiac surgery [3, 4]. In addition, oxygenation and upregulation of neuroglobin (Ngb) and hypoxia-inducible factor-1α (HIF-1α) expression have been considered as a protective mechanism responding to ischemic injury to neurons, while neuron-specific enolase (NSE) is a marker of neuronal damage. Therefore, the circulating levels of Ngb, HIF-1α, and NSE have been used as indicators for cerebral injury [5, 6]. However, whether circulating Ngb, HIF-1α, and NSE levels could serve as early indicators for cognitive dysfunction after cardiac surgery has not been reported.

With the increasing understanding of the mechanisms involved, various treatments have been introduced over the last decade. Shenmai injection (SMI), a Chinese traditional medicine mainly consisting of the two herbal components Radix ginseng Rubra and Radix ophiopogonis, is extensively used in China as an organ protector [7]. Our previous studies [7–10] have demonstrated the protective effects of SMI against oxidative injury and the inflammatory response, showing its potential as a therapeutic agent for the prevention of lung injury after cardiopulmonary bypass (CPB). However, reports concerning the effects of SMI on cognitive function in patients undergoing cardiac valve replacement under CPB are not available.

Therefore, this prospective, randomized, controlled trial was designed to evaluate the predictive effects of serum Ngb, HIF-1α, and NSE on POCD after CPB as well as the protective effect provided by SMI administration in patients undergoing cardiac valve replacement under CPB. The Beijing version of the Montreal Cognitive Assessment (MoCA-BJ; range, 0–30) was analyzed for assessing the cognitive function; and blood gas parameters of the jugular veins as well as Ngb, HIF-1α, and NSE expression levels were used to evaluate cerebral injury.

## Methods

### Study design and patients

This prospective, randomized, controlled trial was conducted from September 2014 to January 2017 at the First Affiliated Hospital of Wenzhou Medical University. This trial was registered with Clinicaltrials.gov as ChiCTR-TRC-14004373 on March 11, 2014. After approval by the hospital’s Clinical Research Ethics Committee and written informed consent from the patients, 90 patients receiving cardiac valve replacement under CPB were screened. This study was reported according to the CONSORT Extension for Chinese Herbal Medicine Formulas 2017: Recommendations, Explanation, and Elaboration checklist [11].

Inclusion criteria included American Society of Anesthesiologists physical status II–III, aged 40–69 years old, body mass index of 18–29 kg/m^2^, left ventricular ejection fraction ≥0.3, and preoperative MoCA-BJ score ≥ 26. Patients were excluded from this study if they had a diagnosis of diabetes mellitus, hypertension, peripheral vascular disease, pulmonary disease, neurological disease, mental disorder, renal insufficiency, liver dysfunction, infective endocarditis, or previous coronary heart disease before screening. In addition, patients who received SMI treatment within 6 months were also excluded.

### Surgical interventions

At 30 min before surgery, 0.2 mg/kg morphine and 0.3 mg/kg scopolamine were given intramuscularly. After a standard monitor was attached, a peripheral venous access was secured. Before induction, a 20-G arterial catheter was cannulated, and the sensor/transducer (FloTrac) was connected to record the waveform. Anesthesia was induced intravenously with 0.01 mg/kg midazolam, 0.6 μg/kg sufentanil, 0.3 mg/kg etomidate, and 0.15 mg/kg vecuronium bromide, and then tracheal intubation was facilitated. The patients received intermittent positive-pressure ventilation with a tidal volume of 8–10 mL/kg and a respiratory rate of 10 beats per min to maintain the end-tidal carbon dioxide pressure at approximately 35 mmHg. Continuous administration of sevoflurane and propofol combined with intermittent injection of sufentanil and vecuronium were used to maintain the depth of anesthesia, which was monitored by the bispectral index. After anesthesia, the right internal jugular vein was reversely cannulated to the bulbar level for continuous blood sampling.

All patients received standard CPB management. CPB was instituted with a membrane oxygenator. The body temperature was maintained under mild hypothermia (32–33 °C), and α-stat was used for acid-base management. The pump flow was maintained at a rate of 2.0–2.5 L/min/m^2^ using a nonpulsatile flow, and a blood cardioplegic solution was used. During the perioperative period including CPB, the mean arterial blood pressure was maintained at 55–80 mmHg using norepinephrine or vasopressin. Milrinone was used if the left ventricular ejection fraction was less than 30% after CPB, as measured by transesophageal echocardiography, in case of right ventricular dysfunction or pulmonary hypertension. A blood transfusion was needed if the hematocrit level fell below 21% during CPB or below 25% during the remaining perioperative period. Fresh frozen plasma was transfused when the international normalized ratio was greater than 1.5 with excessive bleeding greater than 200 mL/h for two consecutive hours in the postoperative period. Platelet concentrates were transfused when the platelet count was less than 50,000/mm^3^ with excessive bleeding greater than 200 mL/h for two consecutive hours in the postoperative period. All patients were transferred to the Coronary Care Unit (CCU) after surgery and received standard management according to institutional guidelines by the CCU staff.

### SMI administration

SMI composed of Radix ginseng Rubra and Radix Ophiopogonis was a product of Sanjiu Pharmaceutical Co. Ltd. (Yaan, China; batch No. Z51021845, provided as 1 g of crude drug in an ampoule of 10 mL). In group S, SMI at a dose of 0.6 mL/kg, dissolved in 250 mL of normal saline, was administered intravenously from the time of anesthesia induction to the beginning of CPB at a rate of 10 mL/min. The control patients were given an equal volume of normal saline instead.

### Outcome measures

The durations of aortic cross clamping, CPB, and anesthesia maintenance were recorded, respectively. The hemodynamic parameters, including the mean arterial blood pressure and heart rate, as well as the blood gas parameters of the jugular veins, which included the jugular venous oxygen saturation (S_J_vO_2_), jugular venous oxygen partial pressure (P_J_vO_2_), hemoglobin, hematocrit, and lactic acid (Lacjv) of the jugular venous blood samples, were collected at 30 min after induction (T_0_), immediately after the endonasal temperature rewarmed to 36 °C (T_1_), the end of cardiac surgery (T_2_), and 6 h (T_3_), 24 h (T_4_), 48 h (T_5_), and 72 h (T_6_) after CPB, respectively. The MoCA-BJ scores were evaluated at 3 days before surgery as well as 3 days, 7 days, and 1 month after surgery.

The remaining blood sample was centrifuged to separate the serum, which was then stored at − 20 °C for subsequent analyses of Ngb, HIF-1α, and NSE. Indicators were determined using commercially available enzyme-linked immunosorbent assay kits (Westang Biotechnology Co. Ltd., Shanghai, China), according to the manufacturer’s instructions. The levels of these indicators were measured by investigators unaware of the group allocation and the blood gas results.

### Sample size calculation and randomization

The sample size was planned and calculated according to comparison of the MoCA-BJ on the third and seventh day postsurgery. With a power of 66% and a significance level of 5%, a total of 17 patients would be required in each group to make a difference of − 3.4 in the MoCA-BJ on the third day postsurgery, with a dropout rate of 10%. With a power of 31% and a significance level of 5%, a total of 28 patients would be required in each group to make a difference of − 1.7 in the MoCA-BJ on the seventh day postsurgery, with a dropout rate of 10%. Thus, enrollment of 88 patients was planned to minimize statistical errors.

Patients were randomized using a sealed envelope system. Eighty-eight patients were randomized to either the SMI (group S, 44 cases) or the control (group C, 44 cases) group. The medical staff collecting and analyzing the MoCA-BJ scores was blinded to the treatment allocation.

### Statistical analysis

Data were tested for normal distribution using the Kolmogorov–Smirnov test. Continuous, normally distributed data were expressed as the mean ± standard deviation. Categorical data were expressed as numbers. Data on the block level were expressed as a median (range) and analyzed with the Mann–Whitney test. For comparison of demographic and operative data, categorical data were compared with the χ^2^ test and quantitative data were compared with the Student’s t-test. For comparison of outcomes within the same group at different time points, data were analyzed by repeated-measures analysis of variance using the Bonferroni method. For comparison of outcomes between different groups at the same time point, the paired t-test was conducted. A *P* value < 0.05 was considered significant. Analysis was performed using SPSS software, version 17.0 for Windows.

## Results

### Patient enrollment and characteristics

**Fig. 1:**
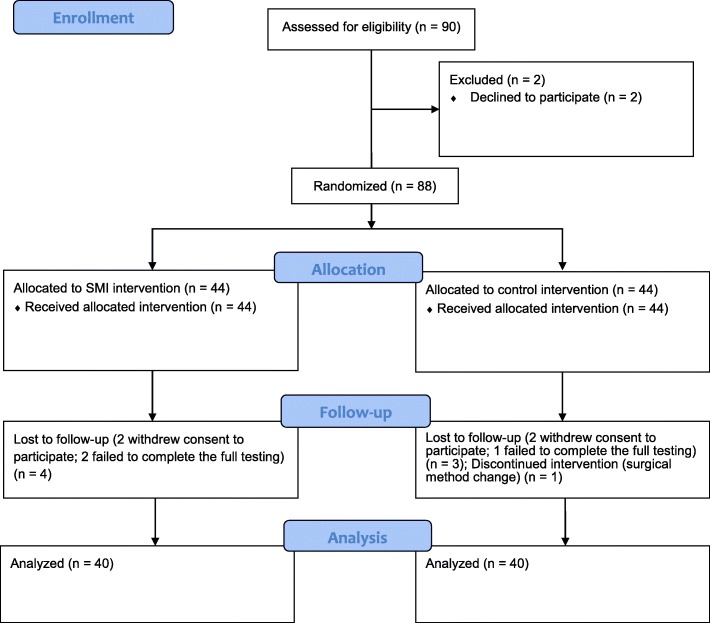
CONSORT 2010 Flow Diagram

The study flow diagram is shown in Fig. 1. A total of 80 patients completed the study. No significant differences between the two groups were found in terms of the clinical characteristics, including gender, age, body mass index, surgical procedure type, duration of aortic cross clamping, CPB, and anesthesia maintenance (*p* > 0.05) (Table 1).

**Table 1:** Clinical Characteristics of Patients (*n* = 40 in Each Group)

	Group C	Group S
Gender (M/F)	23/17	22/18
Age (yr)	55 ± 10	58 ± 9
Body mass index (kg/m2)	23.0 ± 2.8	24.2 ± 2.4
Valve replacement(n)		
single valve replacement	24	26
double valve replacement	16	14
Duration of ACC (min)	89 ± 21	86 ± 20
Duration ofCPB(min)	116 ± 22	111 ± 24
Duration of anesthesiamaintenance (min)	247 ± 33	239 ± 29

*ACC* aortic cross clamp, *CPB* cardiopulmonary bypass

Data are presented as mean ± SD, or numbers

### Hemodynamic parameters

In comparison to T_0_, the mean arterial blood pressure was decreased at T_1_ (*p* < 0.001) and increased at T_4,6_ (*p* = 0.005 and *p* = 0.041) in group C, while it was decreased at T_1,3_ (*p* < 0.001 and *p* = 0.021) and increased at T_4–6_ (*p* = 0.002, *p* = 0.002, and *p* = 0.006) in group S. The heart rate was significantly decreased at T_1_ and increased at T_2–6_ in both groups (*p* < 0.001). There were no differences in hemodynamic variables (mean arterial blood pressure and heart rate) at any time points (*p* > 0.05) between these two groups (Table 2).

**Table 2:** MAP and HR (n = 40 in Each Group)

	Group	T_0_	T_1_	T_2_	T_3_	T_4_	T_5_	T_6_
MAP	C	73 ± 10	60 ± 6^*^	71 ± 9	71 ± 8	83 ± 8^*^	80 ± 9	80 ± 9^†^
(mm Hg)	S	74 ± 7	62 ± 6^*^	71 ± 8	69 ± 5^†^	81 ± 9^*^	83 ± 9^*^	80 ± 6^*^
HR	C	64 ± 13	0^*^	86 ± 10^*^	91 ± 11^*^	81 ± 9^*^	85 ± 7^*^	80 ± 11^*^
(bpm/min)	S	62 ± 10	0^*^	88 ± 10^*^	93 ± 9^*^	83 ± 11^*^	85 ± 7^*^	81 ± 10^*^

T_0_, 30 min after the induction; T_1_, immediately after temperature rewarmed to 36 °C; T_2_, end of cardiac surgery; T_3–6_, 6 h, 24 h, 48 h, 72 h after CPB

Data are expressed as mean ± SD

* *p* < 0.01 vs. **T**_**0**_.† *p* < 0.05 vs. **T**_**0**_

### Blood gas analysis

**Fig. 2:**
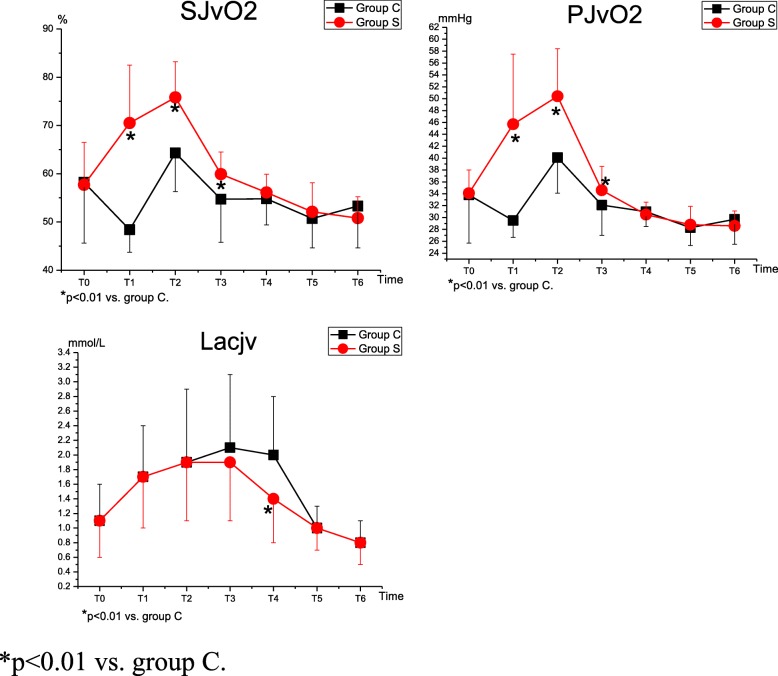
Blood Gas Parameters

Table 3 shows the blood gas parameters. Compared to the baseline values at T_0_, the levels of S_J_vO_2_ and P_J_vO_2_ were increased significantly at T_2_ (*p* = 0.001 and *p* < 0.001) in group C and T_1–2_ (*p* < 0.001) in group S; the values were much higher in group S at T_1–3_ than in group C (*p* < 0.001) (Fig. 2). The levels of Lacjv were increased at T_1–4_ (group C: *p* < 0.001, *p* < 0.001, *p* < 0.001, and *p* = 0.003 and group S: *p* < 0.001, *p* < 0.001, *p* < 0.001, and *p* = 0.002) and decreased at T_6_ (group C: *p* = 0.003; group S: *p* = 0.001) in both groups, compared with T_0_; however the level at T_4_ in group S was lower than that in group C (*p* < 0.001) (Fig. 2). The levels of hemoglobin and hematocrit were lower at T_1–6_ than at T_0_ (*p* < 0.001), but no differences were found between the two groups (*p* > 0.05).

**Table 3:** Blood Gas Parameters (n = 40 in Each Group)

	Group	T_0_	T_1_	T_2_	T_3_	T_4_	T_5_	T_6_
SJ vO2	C	58.2 ± 12.6	48.4 ± 4.7^*^	64.3 ± 8.0^*^	54.7 ± 8.9	54.8 ± 5.4	50.7 ± 6.1^*^	53.3 ± 8.7
(%)	S	57.7 ± 8.8	70.5 ± 12.0^*‡^	75.8 ± 7.4^*‡^	59.9 ± 4.6^‡^	56.1 ± 3.8	52.1 ± 6.0^*^	50.8 ± 4.4^*^
PJ vO2	C	33.8 ± 8.1	29.5 ± 2.8^*^	40.1 ± 6.0^*^	32.1 ± 5.1	31.0 ± 2.5^†^	28.3 ± 3.0^*^	29.7 ± 4.2^†^
(mmHg)	S	34.1 ± 3.9	45.7 ± 11.8^*‡^	50.4 ± 8.0^*‡^	34.6 ± 4.0^‡^	30.5 ± 2.1^*^	28.8 ± 3.1^*^	28.6 ± 2.5^*^
Lacjv	C	1.1 ± 0.5	1.7 ± 0.7^*^	1.9 ± 1.0^*^	2.1 ± 1.0^*^	2.0 ± 0.8^*^	1.0 ± 0.3	0.8 ± 0.3^*^
(mmol/L)	S	1.1 ± 0.5	1.7 ± 0.7^*^	1.9 ± 0.8^*^	1.9 ± 0.8^*^	1.4 ± 0.6^*‡^	1.0 ± 0.3	0.8 ± 0.3^*^
Hb	C	12.8 ± 1.4	8.1 ± 0.9^*^	9.5 ± 1.1^*^	10.5 ± 1.3^*^	11.1 ± 1.3^*^	9.9 ± 1.1^*^	9.8 ± 1.1^*^
(g/dL)	S	13.0 ± 1.1	8.1 ± 0.7^*^	9.6 ± 1.0^*^	10.3 ± 0.9^*^	11.3 ± 0.9^*^	10.1 ± 1.3^*^	10.2 ± 1.4^*^
HCT	C	37.8 ± 4.1	24.0 ± 2.8^*^	28.0 ± 3.1^*^	30.9 ± 3.2^*^	32.5 ± 3.9^*^	29.2 ± 3.3^*^	28.7 ± 3.1^*^
(%)	S	38.1 ± 3.3	24.0 ± 1.9^*^	28.3 ± 3.0^*^	30.4 ± 2.6^*^	33.2 ± 2.7^*^	29.7 ± 3.9^*^	30.1 ± 4.0^*^

T_0_, 30 min after the induction; T_1_, immediately after temperature rewarmed to 36 °C; T_2_, end of cardiac surgery; T_3–6_, 6 h, 24 h, 48 h, 72 h after CPB

Data are expressed as mean ± SD

* *p* < 0.01 vs. **T**_**0**_.† *p* < 0.05 vs. **T**_**0**_.‡ *p* < 0.01 vs. group C

### Concentrations of Ngb, HIF-1α, and NSE

**Fig. 3:**
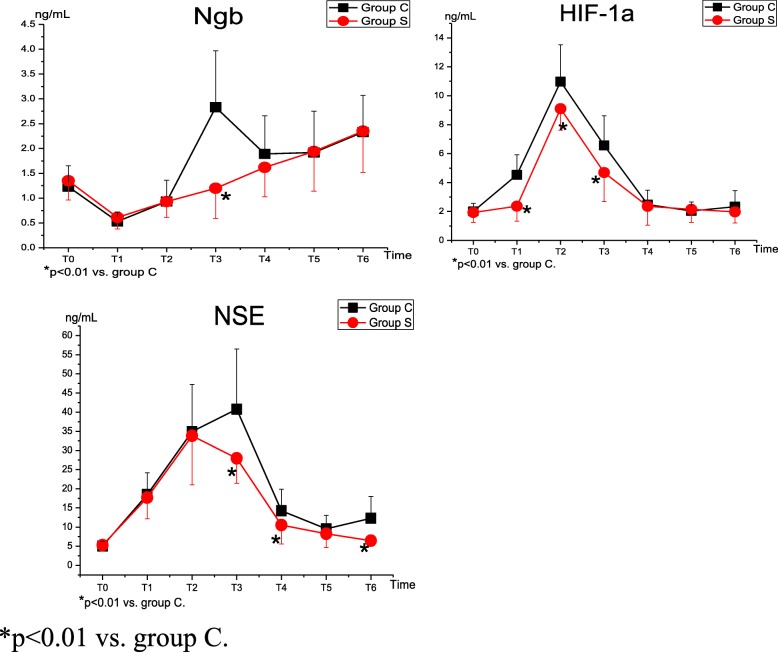
Serum Levels of Ngb, HIF-1α and NSE

Table 4 shows the serum levels of Ngb, HIF-1α, and NSE in both groups. Compared with T_0_, the levels of serum Ngb in group C were significantly decreased at T_1–2_ (*p* < 0.001 and *p* = 0.004) and significantly increased at T_3–6_ (*p* < 0.001), with two peaks at T_3_ and T_6_, respectively; while the levels in group S were significantly decreased at T_1–2_ (*p* < 0.001) and significantly increased at T_4–6_ (*p* = 0.023, *p* < 0.001 and *p* < 0.001), with the highest levels at T_6_. The levels of serum HIF-1α were increased at T_1–4_ and had peaks at T_2_ in both groups (group C: *p* < 0.001, *p* < 0.001, *p* < 0.001, and *p* = 0.001; group S: *p* = 0.001, *p* < 0.001, *p* < 0.001, and *p* = 0.023). The serum NSE levels in both groups were significantly increased at T_1–6_ (*p* < 0.001). The serum levels of Ngb at T_3_ (*p* < 0.001), HIF-1α at T_1–3_ (*p* < 0.001), and NSE at T_3–4,6_ (*p* < 0.001, *p* = 0.002, and *p* < 0.001) were lower in group S than in group C (Fig. 3).

**Table 4:** Serum Levels of Ngb, HIF-1α and NSE(n = 40 in Each Group)

	Group	T_0_	T_1_	T_2_	T_3_	T_4_	T_5_	T_6_
Ngb	C	1.23 ± 0.42	0.53 ± 0.19^*^	0.93 ± 0.43^*^	2.83 ± 1.14^*^	1.89 ± 0.77^*^	1.92 ± 0.83^*^	2.33 ± 0.74^*^
(ng/mL)	S	1.35 ± 0.39	0.61 ± 0.23^*^	0.93 ± 0.32^*^	1.20 ± 0.61^‡^	1.62 ± 0.59^†^	1.94 ± 0.80^*^	2.35 ± 0.83^*^
HIF-1α	C	2.01 ± 0.55	4.54 ± 1.38^*^	10.98 ± 2.55^*^	6.56 ± 2.06^*^	2.48 ± 0.99^*^	2.04 ± 0.62	2.33 ± 1.12
(ng/mL)	S	1.93 ± 0.68	2.37 ± 1.04^*‡^	9.11 ± 1.49^*‡^	4.69 ± 2.01^*‡^	2.36 ± 1.30^†^	2.15 ± 0.90	1.98 ± 0.77
NSE	C	5.05 ± 1.21	18.47 ± 5.72^*^	34.93 ± 12.33^*^	40.77 ± 15.73^*^	14.25 ± 5.67^*^	9.54 ± 3.50^*^	12.27 ± 5.76^*^
(ng/mL)	S	5.28 ± 1.35	17.67 ± 5.51^*^	33.82 ± 12.78^*^	27.96 ± 6.53^*‡^	10.51 ± 4.94^*‡^	8.25 ± 3.61^*^	6.44 ± 1.35^*‡^

T_0_, 30 min after the induction; T_1_, immediately after temperature rewarmed to 36 °C; T_2_, end of cardiac surgery; T_3–6_, 6 h, 24 h, 48 h, 72 h after CPB

Data are expressed as mean ± SD

* *p* < 0.01 vs. T_0_.† *p* < 0.05 vs. T_0_.‡ *p* < 0.01 vs. group C

### MoCA-BJ analysis

**Fig. 4:**
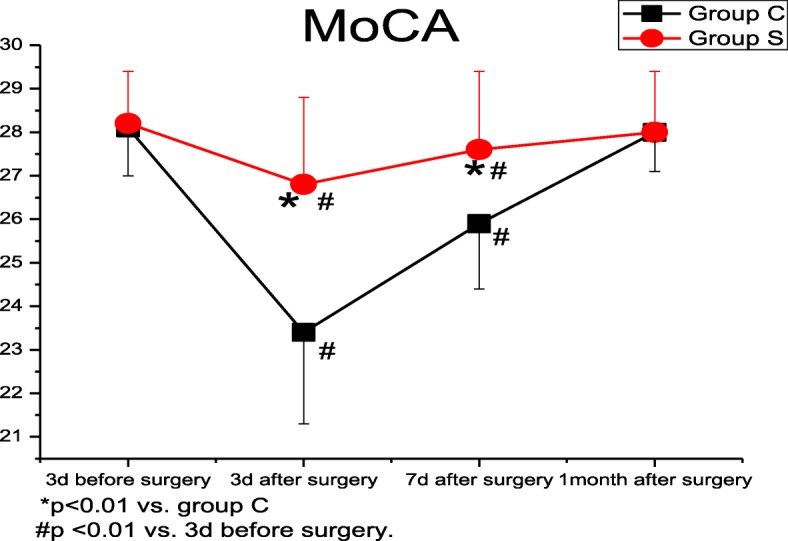
Total MoCA-BJ scores

**Fig. 5:**
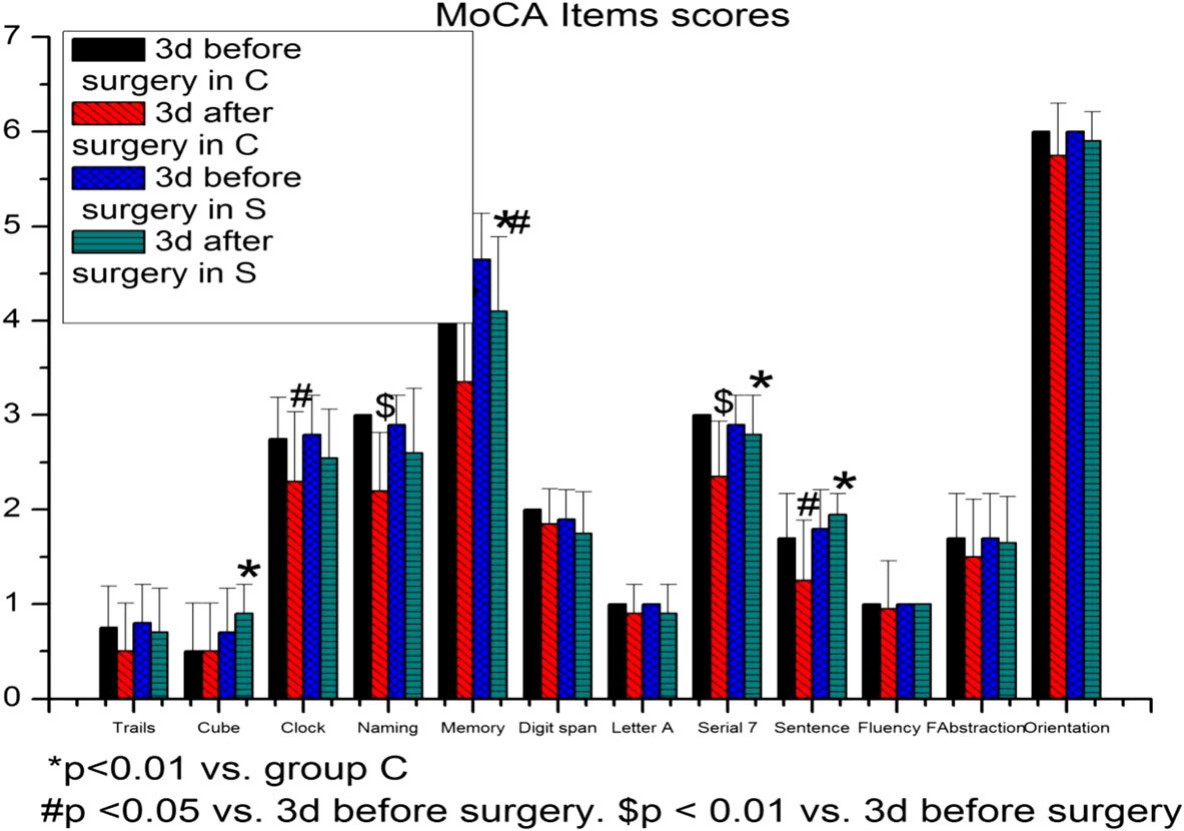
MoCA-BJ Items scores

Compared to preoperative values at 3 days before surgery, the MoCA-BJ scores were decreased at 3 and 7 days after surgery in both groups (group C: *p* < 0.001; group S: *p* < 0.001, *p* = 0.001). The MoCA-BJ scores of group S were higher than those in group C at 3 and 7 days after surgery (*p* < 0.001) (Fig. 4). The scores of Clock, Naming, Memory, Serial 7, and Sentence rep in group C (*p* = 0.025, *p* < 0.001, *p* < 0.001, *p* < 0.001 and *p* = 0.015) and the score of Memory in group S (*p* = 0.012) were lower than the preoperative baseline values at 3 days after surgery. Compared to group C, the scores of Cube, Memory, Serial 7, and Sentence rep were elevated at 3 days after surgery in group S (*p* = 0.005, *p* = 0.005, *p* = 0.008, and *p* < 0.001) (Fig. 5).

## Discussion

Recent technological advances have contributed to an increasingly lower rate of clinically evident complications, but POCD remains an important clinical problem associated with cardiac surgery [12, 13]. Its manifestations may include impairment in visuospatial and executive functions, naming, attention, language, delayed recall (memory), and orientation [14]. Presently, the MoCA and the Mini-Mental State Examination (MMSE) are the most commonly used neuropsychological tests for POCD. As a simple cognitive screening tool, the MoCA-BJ was used in this study to balance the influences of various ages and education levels; [15] and its sensitivity and specificity are better than those of the MMSE [16, 17]. As shown in this study, the total MoCA-BJ scores were significantly lower at 3 and 7 days after surgery in patients receiving cardiac surgery in group C, and the scores basically recovered to baseline values at 1 month after surgery, suggesting that the patients suffered from POCD in the early but not the late stage after cardiac surgery. The results also showed that several cognitive domains, especially memory, were badly impaired; these results were identical to previous ones [18, 19]. For example, Yu et al. [16] have reported that among all the cognitive subdomains, delayed recall (Memory) was shown to be the best index to differentiate POCD from the normal controls. Similarly, Jones et al. [20] have reported that the severity of heart/circulation problems independently contributed to a worse delayed recall (Memory) performance. Our results showed that the Memory scores were lower than the baseline values at 3 days after surgery in both groups, but the scores in group S were improved after SMI administration. In addition, changes in the Clock, Naming, Serial 7, and Sentence rep scores were similar as the delayed recall evaluation, which were all lower than the preoperative baselines values.

Though the cognitive damages after cardiac surgery have been well described, the exact mechanisms involved have not been elucidated. Zheng et al. [21] have reported an association between decreased regional cerebral oxygen saturation (rScO_2_), desaturation, and POCD in adult patients receiving cardiac surgery. The blood flow of the internal jugular vein bulbar is mainly from the cerebral hemisphere, and the levels of S_J_vO_2_ and P_J_vO_2_ can indirectly reflect rScO_2_ [22, 23]. As shown in our study, compared to the baseline values, the levels of S_J_vO2 and P_J_vO2 were significantly decreased and the levels of Lacjv were increased in patients undergoing CPB, suggesting that cerebral anoxia may at least partly contribute to POCD.

Ngb, a novel neuroprotective protein, affords protection against hypoxia/ischemia and oxidative stress-induced injury in the nervous system. Ngb overexpression enhances cell survival under conditions of anoxia or oxygen and glucose deprivation [24], and it protects neurons against cerebral ischemia-reperfusion injury [25]. Previous data have implied that the upregulated expression of Ngb could be an endogenous compensatory or protective mechanism in response to sublethal hypoxic/ischemic insults to brain neurons, and Barzo et al. [26] have reported that a predominantly vasogenic edema formation occurred immediately after brain injury and a more widespread and slower cytotoxic edema formation resulted later, which may lead to two peaks of Ngb expression following brain injury. Furthermore, recent studies have found that the Ngb levels are increased in early and moderately advanced Alzheimer’s disease subjects [27]. These findings may explain the results uncovered in our study.

HIF-1α has emerged as a critical oxygen-sensitive transcription factor that orchestrates the body’s protective response to hypoxia and can protect organs against acute ischemic injury [28]. Moreover, it has been recently documented that the expression of HIF-1α under hypoxia has protective effects on astrocytes, thus playing an important role in cerebral protection [29]. Its protective effects also have been proven in a model of cultured cortical neurons exposed to moderate hypoxia [30, 31]. As shown in our study, the serum HIF-1α level began to increase before the Ngb level. The role of HIF-1α in the regulation of oxygen homeostasis in tissue may be correlated with Ngb expression [32]. Additionally, a recent study has demonstrated that HIF-1α contributes to the upregulation of Ngb expression under hypoxic conditions in mice [33]. However, further studies are necessary to elucidate the exact mechanisms of HIF-1α in Ngb regulation under hypoxic and other pathological conditions.

NSE, which was originally described by Moore and McGregor in 1965, [34] has been considered as a traditional marker to assess neuronal damage; [35] however, clinical trials have shown controversial results regarding the correlation between cognitive dysfunction and the circulating NSE level. Some studies have found a correlation between the NSE level and the clinical outcome of neurocognitive dysfunction, [36–38] but others have failed to do so [39, 40]. In our study, the serum NSE levels were significantly increased after CPB. Therefore, NSE may be an indicative parameter for POCD.

SMI, a traditional Chinese herbal medicine, is widely used in mainland China for the treatment of cardio/cerebrovascular disorders and as an adjunct therapy to tumor chemotherapy [41]. The active component of SMI is radix ginseng rubra, which contains Ginsenoside Rg3 and Ginsenoside Rb1. The protective effects of Ginsenoside Rb1 have been well proven in various models of cerebral ischemia-reperfusion injury [42, 43]. Its possible mechanism of the protective effect on the central nervous system involves calcium channel blockade, estrogen-like action, and antiperoxidation, which may inhibit cerebral nerve cell apoptosis and ameliorate mitochondrial dysfunction, etc [44]. Hence, we hypothesized that treatment with SMI might present beneficial effects against reperfusion injury and improve cognitive function after cardiac valve replacement under CPB. As shown in our study, even though the MoCA-BJ score in group S was decreased at 3 days after surgery but was still improved as compared to group C at the early stage after CPB, SMI treatment increased the levels of S_J_vO_2_ and P_J_vO_2_ as well as decreased the serum Ngb, HIF-1α, and NSE levels at different time points. These data implied that SMI may attenuate cerebral anoxia and neuronal damage, subsequently improving cognitive function and reducing the incidence of POCD.

Several limitations of this study should be noted. First, enrollment of patients did not consider the traditional Chinese medicine syndromes, and the lack of traditional Chinese medicine syndrome diagnosis during patient enrolment was another potential source of selection bias. Second, whether SMI exerts protective effects by improving cerebral oxygenation directly or by other implied mechanisms should be investigated in future studies. Finally, the optimal dosage of SMI and its main effective ingredient were not determined in the current study. Our future studies will address these issues.

## Conclusions

In conclusion, cognitive function is impaired postoperatively in patients undergoing cardiac surgery with CPB. The novel biomarkers Ngb, HIF-1α, and NSE could serve as early indicators for POCD. Treatment with SMI, a traditional Chinese medicine, decreases the levels of serum Ngb, HIF-1α, and NSE, thus improving cognitive function.

## Abbreviations

CCU: Coronary Care Unit
CPB: Cardiopulmonary bypass
HIF-1α: Hypoxia-inducible factor-1α
MoCA-BJ: Beijing version of the Montreal Cognitive Assessment
Ngb: Neuroglobin
NSE: Neuron-specific enolase
POCD: Postoperative cognitive dysfunction
rScO_2_: Regional cerebral oxygen saturation
SMI: Shenmai Injection
